# Computer-aided classification of indirect immunofluorescence patterns on esophagus and split skin for the detection of autoimmune dermatoses

**DOI:** 10.3389/fimmu.2023.1111172

**Published:** 2023-02-28

**Authors:** Jens Hocke, Jens Krauth, Christopher Krause, Stefan Gerlach, Nicole Warnemünde, Kai Affeldt, Nina van Beek, Enno Schmidt, Jörn Voigt

**Affiliations:** ^1^ Institute for Experimental Immunology, affiliated to EUROIMMUN Medizinische Labordiagnostika AG, Lübeck, Germany; ^2^ Department of Dermatology, Allergology and Venerology, University Hospital Schleswig-Holstein/University of Lübeck, Lübeck, Germany; ^3^ Lübeck Institute of Experimental Dermatology (LIED), University of Lübeck, Lübeck, Germany

**Keywords:** autoimmune dermatoses, neural networks, immunofluorescence tests, tissue classification, deep learning

## Abstract

Autoimmune bullous dermatoses (AIBD) are rare diseases that affect human skin and mucous membranes. Clinically, they are characterized by blister formation and/or erosions. Depending on the structures involved and the depth of blister formation, they are grouped into pemphigus diseases, pemphigoid diseases, and dermatitis herpetiformis. Classification of AIBD into their sub-entities is crucial to guide treatment decisions. One of the most sensitive screening methods for initial differentiation of AIBD is the indirect immunofluorescence (IIF) microscopy on tissue sections of monkey esophagus and primate salt-split skin, which are used to detect disease-specific autoantibodies. Interpretation of IIF patterns requires a detailed examination of the image by trained professionals automating this process is a challenging task with these highly complex tissue substrates, but offers the great advantage of an objective result. Here, we present computer-aided classification of esophagus and salt-split skin IIF images. We show how deep networks can be adapted to the specifics and challenges of IIF image analysis by incorporating segmentation of relevant regions into the prediction process, and demonstrate their high accuracy. Using this semi-automatic extension can reduce the workload of professionals when reading tissue sections in IIF testing. Furthermore, these results on highly complex tissue sections show that further integration of semi-automated workflows into the daily workflow of diagnostic laboratories is promising.

## 1 Introduction

Autoimmune bullous diseases (AIBD) are a highly heterogenous group of autoantibody-driven diseases in which autoantibodies against various proteins of the desmosomes and basement membrane zone (BMZ) of the skin and surface epithelia (1–5). Depending on the antigens involved, blister formation either occurs intra-epidermally in pemphigus diseases or sub-epidermally in pemphigoid diseases. Deciphering the affected structures in this heterogeneous group of disorders is essential for both prognosis and treatment, as immunosuppressive therapy varies according to disease entity. Initial differentiation can be achieved by indirect immunofluorescence (IIF) microscopy on monkey esophagus for pemphigus diseases and primate salt-split skin for pemphigoid diseases as the most sensitive screening methods for autoimmune bullous diseases (6, 7). IIF on monkey esophagus can detect circulating autoantibodies against the epithelial and endomysial autoantigens. IIF on primate salt-split skin discriminates autoantibodies against the BMZ. Additional testing with, for example, recombinant desmoglein 1 (DSG1), DSG3, BP180 (type XVII collagen), BP230, laminin 332, type VII collagen, and deamidated gliadin peptides by IIF, enzyme-linked immunosorbent assay (ELISA), or immunoblot analysis (2–4, 8) can be performed to identify target antigens.

Previously, BIOCHIP^®^ mosaics have been developed that allow the simultaneous detection of different autoantibody specificities on a routine laboratory slide by placing multiple miniature so-called biochips in a single incubation field. In addition to recombinant proteins such as BP180 NC16A and cells recombinantly expressing DSG1, DSG3, BP230, type VII collagen, and laminin 332, tissue substrates such as monkey esophagus and primate salt-split skin can also be used (6, 9–11). The BIOCHIP^®^ technology is increasingly used for routine diagnostics of autoimmune blistering diseases (AIBD) (12–22).

**Figure 1:**
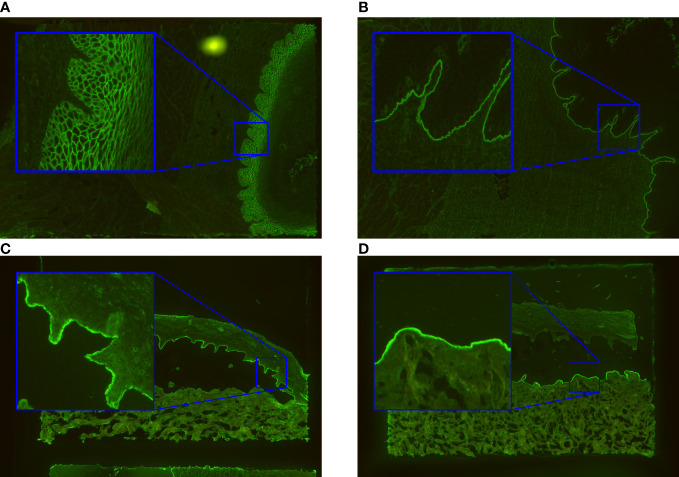
Exemplary immunofluorescence images of the incubated substrates esophagus and salt-split skin. The ‘intercellular’ pattern seen by intercellular labelling of monkey esophagus in pemphigus vulgaris/foliaceus is shown in **(A)**. In **(B)**, the basal membrane zone (‘BMZ’) pattern typical for pemphigoid diseases is indicated. The patterns found in salt-split skin are ‘epidermal’ **(C)** and ‘dermal’ **(D)**. They arise by binding of autoantibodies in pemphigoid patients to the epidermal or dermal side of the artificial split.

Circulating autoantibodies in pemphigus characteristically bind to the epithelium of esophagus in an intercellular pattern (**Figure 1A**). The pattern is seen as smooth linear fluorescence along the borders of the epithelial cells with a mesh-like appearance. The presented algorithm maps this pattern as ‘Intercellular’. Anti-BMZ reactivity in pemphigoid diseases, e.g. directed against BP180, BP230 or type VII collagen can also be visualized in this substrate (**Figure 1B**) revealing to a smooth linear fluorescence along the BMZ. This pattern is subsequently referred to as ‘BMZ’ pattern. Furthermore, in celiac disease and dermatitis herpetiformis, IgA autoantibodies label the endomysium in a characteristic pattern (11) which appears as a honeycombed pattern within the lamina muscularis mucosae. Here, we focused on ‘Intercellular’ and ‘BMZ’ pattern and as such endomysial pattern is not reported. In the diagnosis of AIBDs, other histopathological regions of the esophagus are not relevant.

Separating dermis and epidermis of primate skin with 1M NaCl results in split formation within the lamina lucida, which allows differentiation between two linear IF patterns in pemphigoid diseases. Antibodies against BP180, BP230, and α6β4 integrin stain along the epidermal side of the artificial split (**Figure 1C**). For the computer-generated outputs, we refer to this binding as ‘epidermal’. In contrast, autoantibodies against the p200 antigen, laminin γ1, laminin 332, and type VII collagen bind along the dermal side (23), a pattern that is referred to as “dermal” in this manuscript (**Figure 1D**). In areas where the tissue is still connected, linear fluorescence is seen.

Reading IIF patterns is done by visual evaluation of microscope images and is not standardized. Hence, the results strongly depend on the practical experience of the professional and are subjective. The high variance in the appearance of tissue segments complicates the evaluation leading to an undesirably high variability of the results. Computer-aided evaluation of IIF became popular both because of the automated workflow and more standardized objective evaluation of IIF (24–26). Computer-aided diagnostics rapidly evolved as deep learning became a state-of-the-art method for computer vision tasks. Deep learning enables the solution of complex tasks such as image segmentation (27), object detection (28, 29), and image classification (30). In the medical field, deep learning has been successfully used for the detection of skin cancer (31), lung nodules (32), and diabetic retinopathy (33).

Here, we show how deep learning can be used for computer-aided evaluation of IIF images of highly complex tissue substrates. The images are from slides containing millimeter-sized fragments of coated cover glasses with biological substrates called biochips (EUROIMMUN Medizinische Labordiagnostika AG, Lübeck, Germany). The primary esophagus and primate salt-split skin each cover large areas of the biochip, while only small structures on specific parts of the tissue are decisive for the classification result. Standard deep networks are not suitable for processing these images due to limitations in computer memory and the number of images available for training. We describe two architectures, both of which use segmentation to aid the training process of a classification network. We demonstrate their effectiveness on real data acquired with a EUROPattern^®^ Microscope (EPM) 1.5 microscope (EUROIMMUN) by comparing their outputs with the results of visual evaluation of the same images by a professional.

In the present study, we would also commemorate Detlef Zillikens, director and chair of the Department of Dermatology, University of Lübeck, Lübeck, Germany. Detlef passed away in September 2022 after short and severe disease. He has been one of the leading clinician scientists in the field of AIBD who authored more than 600 articles on this subject. Detlef Zillikens has inspired, motivated, and mentored numerous colleagues including N.v.B. and E.S. E.S. was amongst his first students performing his medical thesis about the presence of inflammatory mediators in sera and blister fluids of bullous pemphigoid patients compared to sera and suction blisters of healthy volunteers in 1993 (34–37). This fruitful and passionate cooperation has been functional for 25 years bringing to light more than 250 articles and book chapters about AIBD. N.v.B. and E.S. are greatly indebted to Detlef Zillikens and thankful for his wise, winning, and kind mentoring and his friendship. All authors are sad and affected about the early death of this exceptional clinician and researcher.

Overall, we present classifiers for the detection and analysis of these IIF patterns. In addition to the classification of circulating antibodies as positive or negative, a brightness score is also returned for titer estimation.

## 2 Materials and methods

### 2.1 Human sera

Serum samples from patients were collected for routine diagnostic analysis by the Immunological Laboratory (Lübeck, Germany) and the Department of Dermatology, University of Lübeck. Samples were anonymized for analysis and stored at -20°C until use. Left-over patient samples were used for this study after completion of all diagnostic procedures. Serum samples from healthy blood donors served as controls. The study performed in accordance with the ethical guideline stated in the Declaration of Helsinki and positively evaluated by the ethics committee of the University of Lübeck (12–178).

### 2.2 Salt-split skin and esophagus dataset

Data were acquired using the EUROPattern^®^ Suite (EUROIMMUN), a system of hardware and software components for computer-aided IIF. Images were acquired using an EPM 1.5 microscope at 10x magnification. To picture an entire biochip, four images are combined. This results in images with slightly varying sizes of about 4,500 x 3,400 pixels.

To generate the training dataset, the biochips were incubated at various dilutions from 1:10 to 1:10,000 to ensure distinction of weak patterns for AIBD from negative samples. The salt-split skin training set consisted of 3,428 images, of which 1,040 images showed a positive reaction in epidermal tissue and 326 showed a positive reaction in dermal tissue. 2,076 of these images showed no positive reaction. The esophagus dataset consisted of 7,022 training images with 1,399 images with intercellular reactivity in the epithelium, 2,539 with anti-BMZ reactivity, and 3,166 images with no or unspecific reactions. The dataset also contained images with patterns caused by a variety of confounder antibodies (e.g., antinuclear antibodies), not associated with AIBD resulting in images with multiple patterns.

To generate the validation dataset, 110 patient samples were incubated at 1:10, 1:32, and 1:100 dilutions to test for correct assignment including weak specific patterns, resulting in three images of esophagus and salt-split skin per patient. For the 52 samples from healthy controls, only a 1:10 dilution was incubated, resulting in one image of esophagus and salt-split skin per control. For the validation dataset, 26 patients with serologically tested epidermal binding to salt-split skin and 8 patients with serologically tested dermal binding were used. 30 patients with serologically tested bullous pemphigoid showed linear binding at the BMZ on monkey esophagus, and 20 patients with serologically tested pemphigus vulgaris/foliaceus stained monkey epithelium with an intercellular pattern. One patient reacted positively to both epidermal BMZ and epithelial desmosomes. In addition, 52 substrates with no or with unspecific reactivity were used, incubated with sera from control subjects or patients with, for example, pemphigus on salt-split primate skin.

The collected training and validation data were read manually by IF professionals. The readers were medical technicians with at least 5 years of experience in reading IF tests including monkey esophagus and salt-split skin.

### 2.3 Salt-split skin algorithm

**Figure 2:**
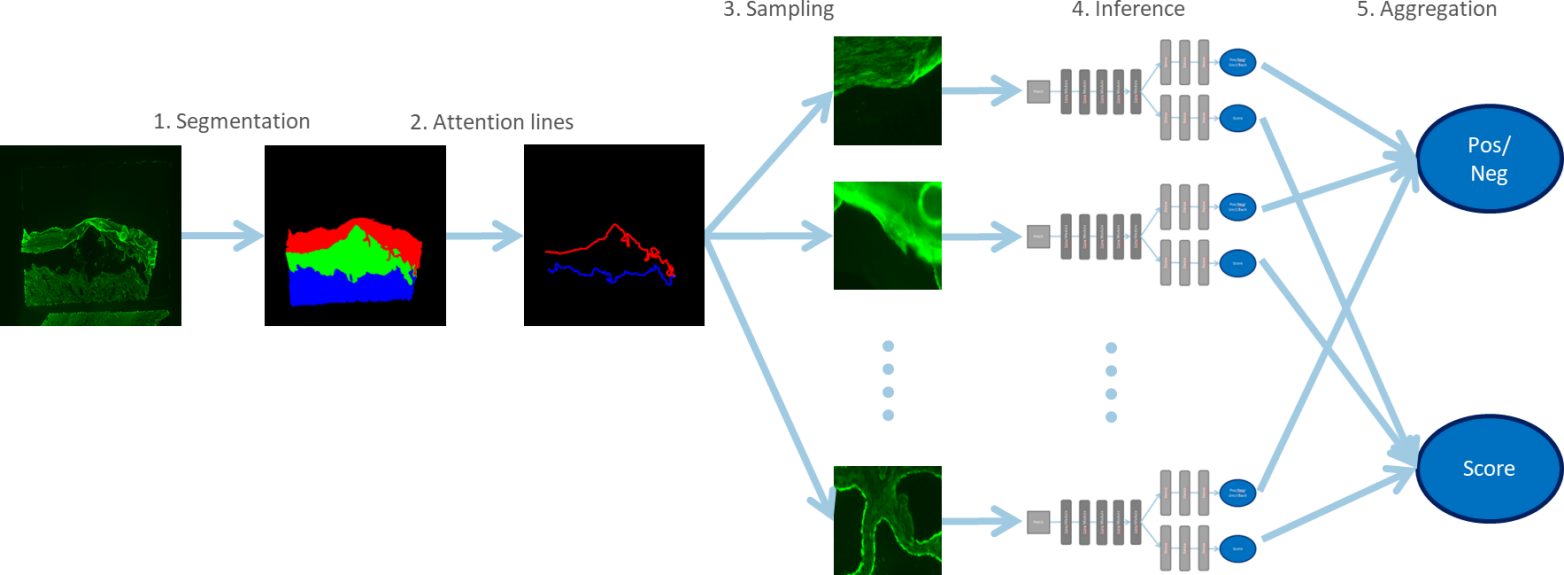
Overview of the steps for processing salt-split skin images. Segmentation is used to identify relevant parts of the image. Samples of these subregions are then analyzed *via* deep networks and the results are aggregated. This approach ensures low memory requirements and good generalization.

The number of salt-split skin images for the training dataset and the memory required to process images of a given size with a deep network is limited. In addition, generalization to unseen data is difficult to achieve due to the large number of parameters needed in a suitable network. Therefore, we chose to first identify relevant subregions of the image and then analyze only those subregions. This approach addresses both memory requirements and generalization. The algorithm includes multiple steps of image processing (**Figure 2**). First, the image is segmented to identify the relevant subregions of the tissue. Based on the segmented regions, lines of attention are computed. Image patches are sampled along these lines and then processed individually by a deep neural network. The individual outputs are aggregated to obtain the final result.

Only the green channel is used for segmentation. The image is downscaled to a resolution of 512 x 512 pixels and then fed into a U-Net type deep network for segmentation. This type of network is known to work well in segmenting microscopy images (27). Here, we used a Fusion-Net, which incorporates recent developments in deep learning and further improves performance (38). The individual outputs are divided into roof (epidermal), floor (dermal), interspace, and background. Here, the interspace is the empty area between the roof and the floor.

The attention regions are then computed. The image content relevant for analysis is located along these attention lines. They are computed from the overlap of the segmented areas expanded by dilation (DIL) (**Figure 2**) by a disc of 3 pixels in diameter. The extended roof (*R*), floor (*F*) and interspace (*I*) subregions are interpreted as sets in the following. The lines *L* on the epidermal (*L_Roof_*) and dermal (*L_Floor_*) substrate are computed as


$$\;L_{Roof}=\text{DIL}\left(R\right)\cap^{}\left(\text{DIL}\left(F\right)\cup^{}\text{DIL}\left(I\right)\right),$$

$$L_{Floor}=DIL\left(F\right)\cap^{}\left(DIL\left(R\right)\cup^{}DIL\left(I\right)\right).$$

To focus on the relevant image content, image patches of size 64 x 64 pixels are extracted along the attention lines. They are taken from a downscaled version of the original image with a size of 2,048 x 2,048 pixels. To achieve a uniform distribution of the patches along the lines, Poisson disc sampling is used (39). For each line, 40 patches are extracted. The following inference steps are performed separately for roof and floor.

**Figure 3:**
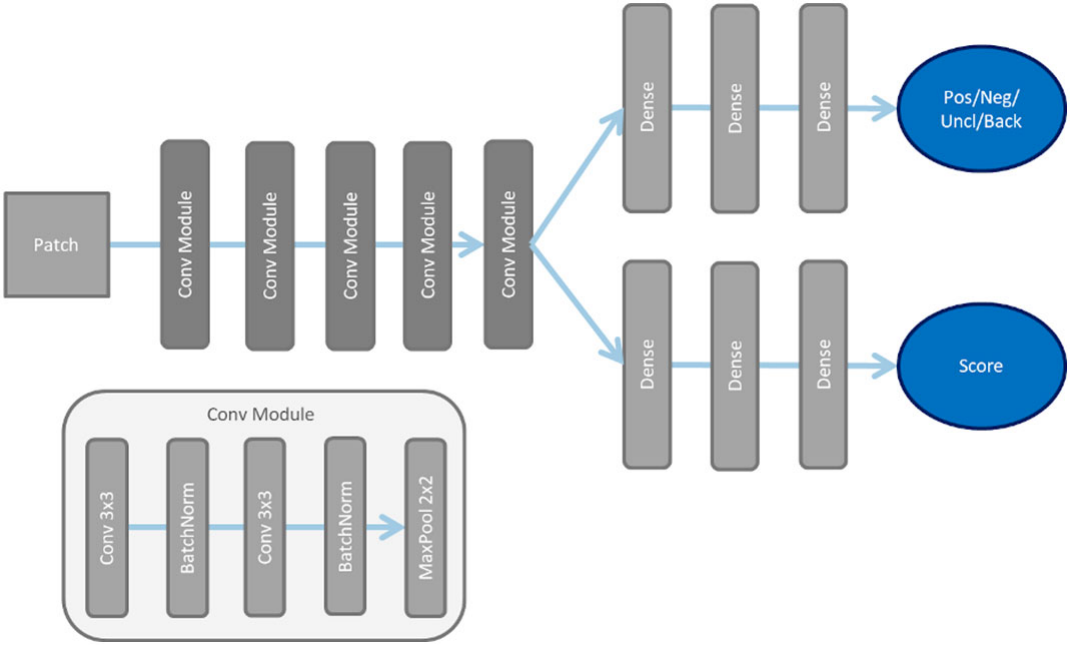
For processing the samples of the subregions of the salt-split skin substrate, the displayed deep network architecture is used. After processing by convolution module shown below, the processing is divided into two branches of dense layers for the different outputs.

Each patch is then processed separately by a deep neural network to obtain local results (**Figure 3**). This is a very small network with only a few layers, so only a few parameters need to be optimized. The network has two outputs: a probability for a label and a brightness score for roof or floor. The label values are ‘positive’, ‘negative’, ‘background’, or ‘unclear’. The ‘unclear’ label is needed because not all sections along the attention lines provide the information needed for classification. The ‘background’ label was only used during training to indicate empty patches.

Finally, the labels of the patches are aggregated to obtain a result for the entire attention line and thus for the entire image. Every patch *x_i_* is processed by a deep network *f*. For every index *i*∈*N* the network returns ‘negative’ as the label with the highest probability. Accordingly, *i*∈*P* if ‘positive’ is the label with highest probability. The other labels ‘background’ and ‘unclear’ are ignored during aggregation. Therefore, these regions do not influence the result. The result *y*∈[0,1] is given by


$$y=\frac{1+y_{pos}-y_{neg}}{2}\;,\;where\;y_{neg}=\frac{\sum_{i\in N}f\left(x_{i}\right)}{\left|N\right|},\;y_{pos}=\frac{\sum_{i\;\in P}f\left(x_{i}\right)}{\left|P\right|}.$$

The brightness *b*∈ℝ is obtained from the mean of the three samples *P*_3_ with the highest probability value for the label ‘positive’:


$$b=\frac{\sum_{i\;\in P_{3}}b_{i,\;}}{\left|P_{3}\right|}.$$

These brightness scores are then combined for images of different dilutions to estimate the titer.

#### 2.3.1 Training of the salt-split skin neural networks

Both the segmentation network and the network for processing patches were optimized using adaptive moment estimation (ADAM) (40). For the segmentation network, a generalized dice loss (41) was used due to its robustness to imbalanced region sizes. The patch analysis network has two outputs, containing classification and regression output. Cross entropy was used to derive the classification output, and mean squared error was used to derive the regression output. Both were weighted equally. Separate analysis networks were trained for roof and floor to achieve a better adaption to the pattern features. In total, there are three networks: one for segmentation, one for the analysis of the roof, and one for the analysis of the floor.

Several augmentations were applied to improve the generalization of these networks. When training the segmentation network, flip and rotation were applied to the entire image. For the analysis networks, the used augmentations flip and Gaussian noise were applied only to the patches.

As mentioned earlier, when training the analysis networks, empty patches are labeled as ‘background’ for technical reasons. It is easier to train the networks with a fixed number of patches per image, but since the size of the salt-split skin segment varies, the number of extracted patches also varies. Therefore, a fixed, generous number of patches is assumed for each image, and missing patches are inserted as empty ones.

### 2.4 Esophagus algorithms

For the diagnosis of AIBDs, the detection of circulating autoantibodies against epidermal/epithelial antigens is essential. Monkey esophagus is the most sensitive tissue substrate for serum autoantibodies in pemphigus vulgaris/foliaceus. In pemphigoid diseases, autoantibodies label the BMZ of monkey esophagus, but with a lower sensitivity compared with primate salt-split skin (15, 42–45). The algorithm for classification of the immunofluorescence (IF) patterns on esophagus tissue is divided into two subtasks.

The first task is to localize and segment the tissue segments in the image. An adapted version of the U-Net model (27) is trained on an esophagus dataset containing image pairs of IF images and segmentation ground truth images. The segmentation images consist of seven segments representing the esophagus tissue sections longitudinal muscle, circular muscle, muscular mucous membrane, BMZ, lamina propria, epithelium, and background. The trained neural network can process unseen IF images of the esophagus and assigns one of the aforementioned sections to each pixel of the image. The key information of the segmentation result is the spatial information of the epidermal BMZ that could be used in the following subtask.

**Figure 4:**
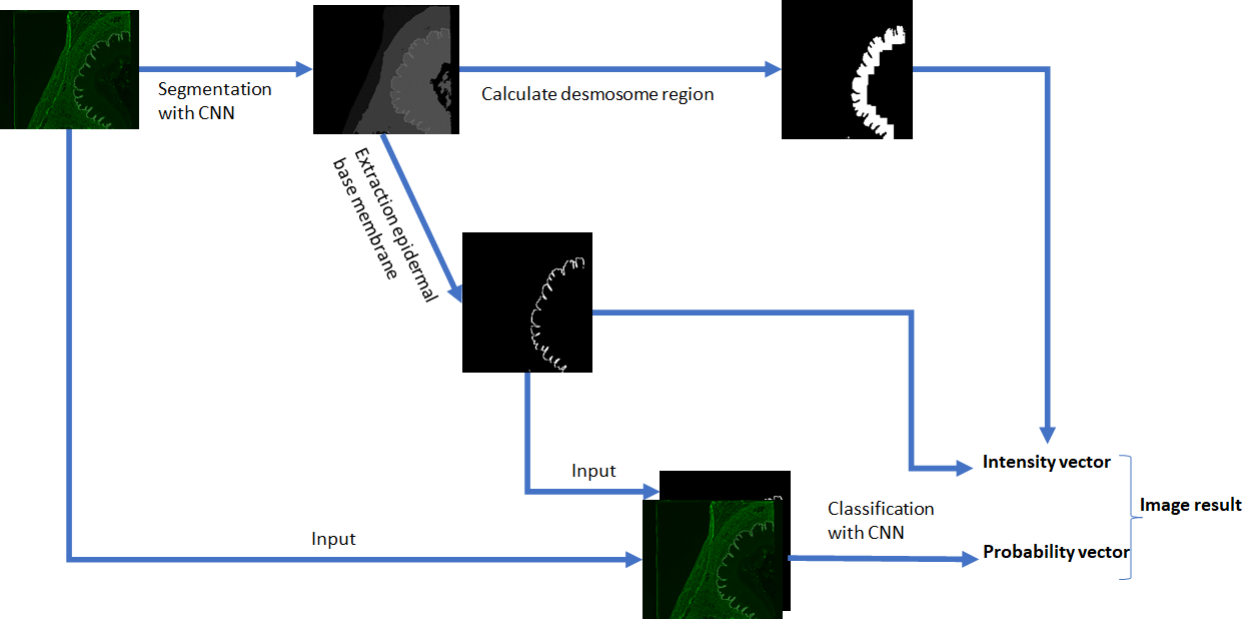
Steps for processing esophagus images. The first processing path segments the image with a convolutional neural network (CNN) into six tissue segments plus background and extracts the epidermal basement membrane as input to the classification path. The segmentation mask is post-processed to calculate the desmosome region. The spatial information is used for intensity estimation of found pattern.

The second task is the classification of the IF image into the classes ‘BMZ’, ‘intercellular’ and ‘negative’. One approach to achieve this goal is a whole-image classification neural network that is trained on a dataset of esophagus IF images and corresponding target labels, assigning a probability between 0 and 1 for each class to each image. However, the neural network had low accuracy in predicting the ‘BMZ’ class. Presumably, the sparse information of the very thin epidermal BMZ compared with the large number of other pixels occurring in the image and the high variance of tissue morphologies prevented the neural network from focusing on the crucial image regions and features. To bypass this disadvantage, the spatial information of the epidermal BMZ processed in the first step can be utilized. The classification neural network is designed to receive two input images: The first input is the original IF image, and the second is a post-processed mask based on the segmentation result. The post-processing drops all segmentation information except the epidermal BMZ and converts the remaining image to a binary image. The binary image is used as attention mechanism input and is intended to hint the neural network to pay special attention to the masked region (**Figure 4**). This approach dramatically increased the accuracy in our classification experiments.

At last, not only the classification with a probability between 0 and 1 for each class, but also a quantification of pattern intensity, which represents the titer of the autoantibodies in patient serum, is valuable information for the evaluation of IIF images, in particular, if more than one pattern is detected. Hence, the algorithm extracts the intensity of the pattern in the regions where the pattern occurs and predicts its titer. For a positive ‘BMZ’ pattern, the intensity extraction is straightforward, when the segmentation information of the esophagus is given. However, for a positive ‘intercellular’ pattern, more post-processing is required to find the relevant region, since the desmosome region in the epithelium is in close proximity to the BMZ and the segmentation contains the epithelium as a whole region. Therefore, post-processing with convolution operations is calculated on the segmentation image to predict the region adjacent to the BMZ that extends into the epithelium (**Figure 1A**). In the case of a positive reaction, the ‘intercellular’ staining appears as the brightest part in that region and can be extracted using a previously defined quantile of the occurring pixel intensities.

#### 2.4.1 Training of the esophagus neural networks

The segmentation neural network for esophagus tissue images was trained on preprocessed and downscaled images of size 512 x 512 pixels. This ensures rapid and memory-optimized segmentation of the images, with resolution high enough to distinguish the different tissue regions that appear. In addition, the images were augmented by horizontal and vertical flipping, random rotation and zooming, resulting in a more generalized network. The segmentation network has seven outputs corresponding to the tissue segments longitudinal muscle, circular muscle, muscular mucous membrane, epidermal basement membrane, lamina propria, epithelium, and background. The network was optimized using the ADAM optimizer and categorical cross-entropy.

The classification neural network was trained on preprocessed images of size 2,048 x 2,048, preserving all the crucial information available in positive reactions. For each image, the previously trained segmentation network processes a segmentation map on the fly, extracting the BMZ, converting it to a binary image and upscales it to the size of 2,048 x 2,048 pixels, building an image input pair of original tissue image and binary map of the epidermal BMZ. The image pairs were also augmented by horizontal and vertical flipping, random rotation and zooming to increase the variance of the training dataset. The classification network also used the ADAM optimizer. The employed optimization function was binary cross-entropy.

### 2.5 Titer estimation

The titer value is computed for the positive IF patterns of each patient to estimate the amount of serum autoantibodies. Titer values are taken from the dilution series(1:10,1:32,1:100,1:320,…). An image of dilution *d_k_* has the k^th^ dilution from that series. For a single image, the brightness score *s*∈[1,5]of a detected pattern is converted to the titer by addition: *d_k_*_+_ *_s_*_-1_. If multiple images are available, the lowest-dilution image with a negative pattern is defining the total dilution. If there is no image with a negative pattern, the highest-dilution image is taken, and the total dilution is computed in the same way as the single image.

## 3 Results

The esophagus and salt-split skin classification algorithms were used to process the samples of the validation dataset. Therefore, each individual image was processed. For sera with several dilutions, each individual image was classified. The identified pattern and intensities were used to compile a patient-based result including the predicted titer. For sera with only one dilution, the result depended solely on the result of that image.

### 3.1 Salt-split skin classification results

**Figure 5:**
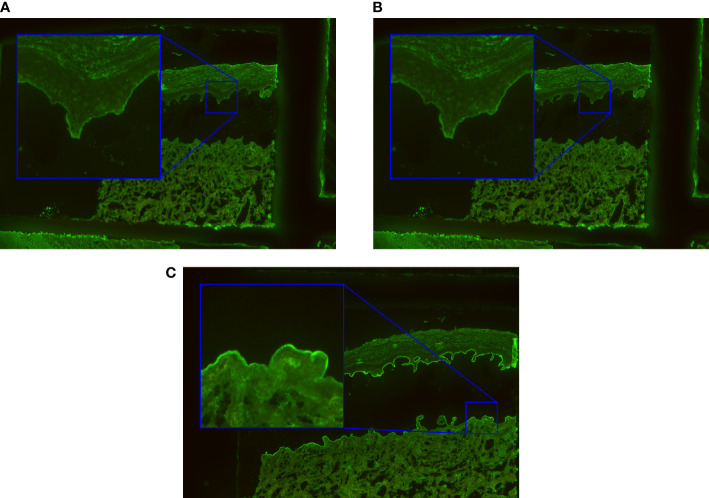
Classification errors usually occur in borderline cases. **(A)** The roof was incorrectly classified as positive due to unspecific immunofluorescence. **(B)** A false negative result was caused by weak staining along the epidermal side of the artificial split. **(C)** The false positive reactivity along the dermal side was the result of unspecific labelling which may occur with strong staining along the blister roof.

The trained salt-split skin models were evaluated using the validation dataset. One patient had to be discarded from this analysis due to a defect in the substrate image. Thus, the validation dataset consisted of 109 patient sera for analysis (**Table 1**). A pattern detection accuracy of 96% was achieved for the salt-split skin ‘epidermal binding’ algorithm. Both positive percent agreement (PPA) and negative percent agreement (NPA) were 96%. Here, the results of one patient serum with a titer of 1:10 gave a barely positive IF result and were incorrectly classified as negative. The salt-split skin ‘dermal binding” algorithm achieved 97% accuracy, with 100% PPA and 97% NPA. Of note, only eight patients showed dermal binding. The classification errors are exemplified in **Figure 5**. These errors occurred mostly in borderline cases where luminance was low and sometimes unspecific. When only ‘binding’ was evaluated, regardless of epidermal or dermal localization, binding was detected with 95% accuracy, with 97% PPA and 95% NPA. Titer estimates were almost all in the +/-1 range for both patterns.

**Table 1:** Performance of the salt-split skin classifier (EPa-Classifier) compared to the conventional evaluation by an IIF professional.

		EUROPattern^®^ visual mode								
EPa Classifier		Combined			‘epidermal’			‘dermal’		
		positive	negative	total	found	not found	total	found	not found	total
	positive	31	4	35	25	3	28	8	3	11
	negative	1	73	74	1	80	81	0	98	98
	total	32	77	109	26	83	109	8	101	109
			Accuracy:	95.4%		Accuracy:	96.3%		Accuracy:	97.3%
			PPA:	96.9%		PPA:	96.2%		PPA:	100.0%
			NPA:	94.8%		NPA:	96.4%		NPA:	97.0%

The combined section shows the overall result when any positive pattern is counted as positive. Samples were present that were positive in both patterns. ‘Epidermal’ and ‘dermal’ indicate reactivities detected along the epidermal and dermal side of the artificial split by IIF on primate salt-split skin.

### 3.2 Esophagus classification results

The algorithm detected 46 of 49 positive patterns. The algorithm classified no patient serum as false positive and 61 patient samples as true negatives. This resulted in an overall agreement of 97%, a PPA of 94% and a 100% NPA (**Table 2**). Of note, 3 sera that were classified as false negative by the algorithm were identified by visual classification as marginally positive with a titer of 1:10.

**Table 2:** Performance of the esophagus classifier (EPa-Classifier) compared to the conventional evaluation by an IIF professional.

		EUROPattern^®^ visual mode								
		Combined			‘BMZ’			‘Intercellular’		
EPa Classifier		positive	negative	total	found	not found	total	found	not found	total
	positive	46	0	46	27	2	29	20	0	23
	negative	3	61	64	3	78	81	0	90	87
	total	49	61	110	30	80	110	20	90	110
			Accuracy:	97.3%		Accuracy:	95.5%		Accuracy:	100.0%
			PPA:	93.9%		PPA:	90.0%		PPA:	100.0%
			NPA:	100.0%		NPA:	97.5%		NPA:	100.0%

The combined section shows the overall result when any positive pattern is counted as positive. Samples were present that were positive in both patterns. The basal membrane zone (‘BMZ’) and ‘intercellular’ pattern reflect IF reactivity against the BMZ and intercellular staining of the epithelium as seen with pemphigoid and pemphigus patients, respectively.

**Figure 6:**
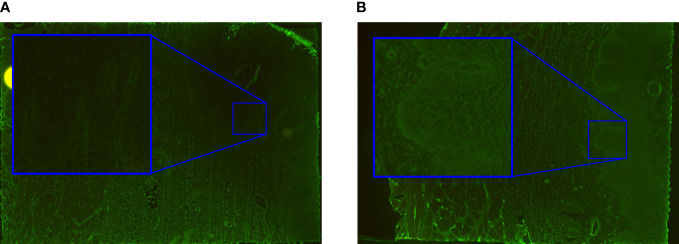
Classification errors on esophagus with faint staining. **(A)** ‘BMZ’ pattern incorrectly classified as ‘negative’. **(B)** ‘Intercellular’ pattern incorrectly classified by the algorithm as ‘BMZ’ in a patient with pemphigoid disease.

**Figure 7:**
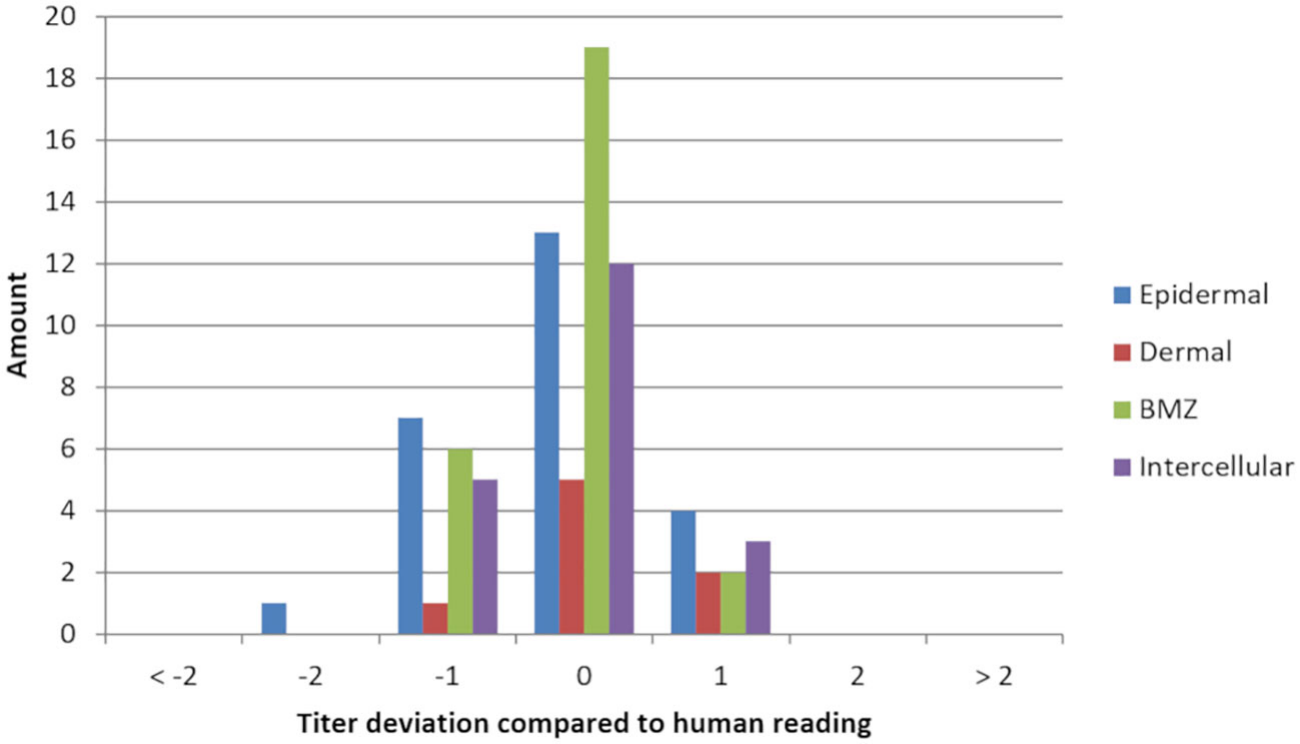
Titer deviation of each pattern compared to the evaluation by an experienced reader of indirect immunofluorescence images. Titer deviation was only calculated in samples where the algorithm and conventional reading detected the same pattern. The reading by experienced personnel was used as a reference. A negative deviation indicates an underestimation of the titer steps, a positive deviation an overestimation by the algorithm.

Regarding the pattern recognition for the ‘BMZ’ pattern, 27 of 30 patient sera were classified by the algorithm as true positive reactions, three patient sera were classified as false negative (the three marginally positive sera, example in **Figure 6A**), and two patient sera were classified with a positive pattern, but instead of a ‘BMZ’ the algorithm predicted an ‘intercellular’ pattern (an example is shown in **Figure 6B**). The algorithm predicted 78 patients as true negative samples to BMZ and two patients as false positive samples. The overall agreement for the pattern is 96% compared to the manual reading with a PPA of 90% and NPA of 98%. The agreement of titer prediction for BMZ, calculated only for the true positive samples, agreed in 70% of patients. 30% of the predictions differed by +/-1 titer step. There were no deviations greater than this (**Figure 7**).

For the ‘intercellular’ pattern, all 20 patient sera were correctly identified by the algorithm and all 90 sera without this pattern were correctly classified as negative pattern. Thus, the overall agreement for the ‘intercellular’ pattern was 100% with a PPA of 100% and NPA of 100%. The titer prediction for the pattern had a 60% match rate for the true positive patient samples. 40% of the titer predictions had an error of +/- 1 titer step. As with the ‘BMZ’ pattern, no deviations greater than this were detected (**Figure 7**).

## 4 Discussion

The diagnostics of AIBD is based on the detection of circulating and tissue-bound autoantibodies, with the latter remaining the gold standard. However, detection of circulating autoantibodies allows further differentiation of disease entities, which is relevant for both prognosis and choice of treatment. IIF testing on monkey esophagus and salt-split skin is commonly used as screening test and provides initial insight into likely disease entities (46). Delayed testing for AIBD and delayed treatment can lead to adverse outcomes with permanent impairment or even fatality (47, 48). While monkey esophagus epithelium has the highest sensitivity for autoantibodies against desmosomal proteins, autoantibodies against BMZ proteins can also be detected (5, 48–50). For the detection of BMZ structures, sensitivity is higher when salt-split skin is used as substrate, which has the advantage that epidermal-binding can also be distinguished from dermal-binding AIBD types (5, 50–52). Analysis of IIF on these substrates requires experienced readers. In this study, automated reading of IIF on primate esophagus and salt-split skin was evaluated. The presented algorithms for classification of both monkey esophagus and salt-split skin tissue sections for the detection of autoantibodies specific for AIBD showed a high accuracy with over 95% agreement compared to the results of conventional reading by an IIF professional. The PPA was above 97% for all positive IF patterns, on both the esophagus and salt-split skin. The NPA was at least 95% for all patterns. The results showed a slightly higher agreement for blister floor staining on salt-split skin and for intercellular staining on monkey esophagus, whereas for blister roof staining on salt-split skin and BMZ staining on monkey esophagus, some samples with low staining intensity were not detected and some samples with unspecific staining were classified as positives.

The confounder autoantibodies described previously were present on both positive and negative samples of the training dataset. The deep learning algorithm was trained to assign a negative label to the patterns caused by the confounder antibodies on images if only confounder patterns are present. When both a cofounder pattern and an AIBD-relevant pattern, i.e. “BMZ”, “intercellular”, “epidermal”, or “dermal” were present, the confounder pattern was suppressed and only the AIBD-relevant pattern indicated. Therefore, the algorithm learned that the patterns caused by confounder antibodies are irrelevant for the desired outcome and can be ignored.

Both algorithms can assist laboratory staff in the challenging task of evaluating IF patterns on tissues. The classifiers are an excellent extension of the screening methods for AIBD. Automation of IIF evaluation has already been successfully used in rheumatology for the detection of antinuclear antibodies. Several commercially available systems can be used here [reviewed in (53)]. In the latter review, the authors conclude that higher standardization of results is achieved by less subjectivity and less influence by expertise and that automated workflows are more efficient due to higher throughput. We think standardization of reading IF patterns, especially tissues can be prone to have a high variance between the readings of different personnel. Computer-aided classification, therefore offers a second opinion in a deterministic manner. However, the limitation of our algorithm lies in the design of a pattern proposal system that only can give hints on learnt patterns. For rare patterns that were not contained in the training dataset, the algorithm will likely fail with its proposal and the IF professional must act independently. Also, our study revealed some discrepancies between the algorithm and the reading of the IF professionals. In these cases, the IF professional must take the final decision. Obviously, the benefit of having a high-throughput systems only takes effect in laboratories with a high number of sera subjected to IIF on esophagus and salt-split skin.

Software solutions for computer-aided diagnostics of IIF are already available for other substrates, using the EUROPattern^®^ Microscope (EUROIMMUN), which was also used in the present study (54). The EUROPattern^®^ Suite software is capable of classifying images of HEp-2/HEp-2010 cells for the detection of anti-nuclear antibodies (ANA) (25, 55), anti-neutrophil cytoplasmatic antibodies (ANCA) (56), anti-mitochcondrial antibody (AMA), anti-Epstein- Barr virus (EBV) antibodies, anti-dsDNA antibodies using *Crithidia luciliae*-based IIF (26), recombinant cell-based assays for multiparametric serological testing in autoimmune encephalitis, e.g., with recombinant cells (54), and rat liver and kidney for detection of reactivity against liver-kidney microsomes (LKM). In the present study, the main challenge was that the small structures relevant for classification were present only on certain parts of the large area covered by the tissue on the biochip. This prevented the use of standard deep networks. We applied segmentation to focus the attention of the classification networks to the crucial regions. For salt-split skin substrate, unspecific and low luminance borderline cases led to the most difficulty because there is no clear decision boundary. The segmentation of esophagus tissue was particularly challenging due to its naturally high variance in tissue layout. Specifically, segmentation and thus detection of the BMZ, which is very thin compared to other sections of the tissue, is a challenging task. Furthermore, classification of the BMZ is complex because it lacks specific structural features. Besides the challenging algorithmic processing, collecting a sufficient amount of training data containing hand-labeled ground truth masks for the segmentation neural network is a tedious and time-consuming manual task.

Our results show that deep learning as a method for computer-aided diagnostics on microscopy images has evolved into a state-of-the-art method besides traditional computer vision. Segmentation and classification of neural networks showed good results in this work. Even very complex microscopy images with tissue layers that are difficult for professionals to evaluate can reliably be segmented and classified *via* computer-aided algorithms.

The present study has several limitations. Only a limited number of sera with salt-split skin dermal binding pattern were applied. Therefore, the accuracy for this pattern is of lower significance. A larger study focusing on the clinical application of the classifiers will certainly improve this aspect and is anticipated. Also, the algorithm only helps to find the expressing patterns for IgG autoantibodies in pemphigus and pemphigoid diseases. Patterns due to IgA autoantibodies are not expected to greatly differ between the ones by IgG reactivity but have not been formally employed in the present study. Currently, there is also no automated assignment of the identified patterns to a specific AIBD. Additional algorithms for advanced diagnostics of AIBD using BIOCHIP^®^ mosaic-based IIF, including classifiers for recombinant BP180 as well as for cell-based IIF with recombinant BP230, DSG1, and DSG3 classifiers, are under development to detect autoantibodies specific for these disorders. The great advantage is that the results of IF image evaluation will be automatically proposed for verification and approval by laboratory personnel. In conclusion, the presented classifiers and algorithms allow the semi-automated assessment of autoantibody binding on monkey esophagus and primate salt-split skin in routine diagnostics of pemphigus and pemphigoid diseases. This innovation will further improve and facilitate the diagnosis of these rare autoimmune disorders.

## Data availability statement

The datasets for this article are not publicly available due to concerns regarding participant/patient anonymity. Requests to access the datasets should be directed to the corresponding author.

## Ethics statement

The studies involving human participants were reviewed and approved by Ethics committee of the University of Lübeck. Written informed consent for participation was not required for this study in accordance with the national legislation and the institutional requirements.

## Author contributions

JK and JH contributed equally to this work and share first authorship. All authors contributed to the article and approved the submitted version.
